# LiDAR Is Effective in Characterizing Vine Growth and Detecting Associated Genetic Loci

**DOI:** 10.34133/plantphenomics.0116

**Published:** 2023-11-17

**Authors:** Elsa Chedid, Komlan Avia, Vincent Dumas, Lionel Ley, Nicolas Reibel, Gisèle Butterlin, Maxime Soma, Raul Lopez-Lozano, Frédéric Baret, Didier Merdinoglu, Éric Duchêne

**Affiliations:** ^1^ INRAE, University of Strasbourg, UMR SVQV, 28, rue de Herrlisheim, 68000 Colmar, France.; ^2^ INRAE, UEAV, 28, rue de Herrlisheim, 68000 Colmar, France.; ^3^ INRAE, Aix-Marseille Université, UMR RECOVER, 3275 Route de Cézanne, 13182 Aix-en-Provence, France.; ^4^ INRAE, Avignon Université, UMR EMMAH, UMT CAPTE, 228, route de l’aérodrome, 84914 Avignon, France.

## Abstract

The strong societal demand to reduce pesticide use and adaptation to climate change challenges the capacities of phenotyping new varieties in the vineyard. High-throughput phenotyping is a way to obtain meaningful and reliable information on hundreds of genotypes in a limited period. We evaluated traits related to growth in 209 genotypes from an interspecific grapevine biparental cross, between IJ119, a local genitor, and Divona, both in summer and in winter, using several methods: fresh pruning wood weight, exposed leaf area calculated from digital images, leaf chlorophyll concentration, and LiDAR-derived apparent volumes. Using high-density genetic information obtained by the genotyping by sequencing technology (GBS), we detected 6 regions of the grapevine genome [quantitative trait loci (QTL)] associated with the variations of the traits in the progeny. The detection of statistically significant QTLs, as well as correlations (*R*^2^) with traditional methods above 0.46, shows that LiDAR technology is effective in characterizing the growth features of the grapevine. Heritabilities calculated with LiDAR-derived total canopy and pruning wood volumes were high, above 0.66, and stable between growing seasons. These variables provided genetic models explaining up to 47% of the phenotypic variance, which were better than models obtained with the exposed leaf area estimated from images and the destructive pruning weight measurements. Our results highlight the relevance of LiDAR-derived traits for characterizing genetically induced differences in grapevine growth and open new perspectives for high-throughput phenotyping of grapevines in the vineyard.

## Introduction

To meet the challenge of adaptation to climate change and the global demand for grapevine varieties resistant to diseases, the number of grapevine genotypes under evaluation is unprecedented and the need for high-throughput phenotyping methods continuously increases [1]. Several teams are developing high-throughput phenotyping systems for this purpose [2,3].

Estimating grapevine growth-related traits is mandatory to appropriately describe the effects of training systems [4,5], management techniques [6–8], environmental conditions [9], or genotypic effects for both rootstocks [10–12] and scions varieties [13].

Grapevine photosynthetic capability is primarily determined by leaf area. A reduced leaf-to-sink ratio can reduce the sugar concentration in berries at harvest [14,15], delay the dates of véraison [16], and impair fruit set and the number of berries per cluster [17]. Leaf area is also a variable requested for estimating the evaporative demand of the plants [18]. Canopy density can also assist in characterizing the microclimate around the clusters and the possible impact of fungal diseases [4,19], and, more recently, in fine-tuning the pesticide dosage [20].

Several methods were proposed to estimate leaf area for the grapevine [21], but they are usually time consuming and difficult to use when a lot of factor levels, as in genetic studies, are to be compared. To characterize grapevine growth capabilities, measuring pruning weight is a popular method because it requires only pruning shears and scales. However, it remains time consuming when hundreds of genotypes are to be characterized.

Multi-spectral and hyperspectral cameras onboard unmanned aerial vehicles (UAVs) and satellites enabled the estimation of vineyards' architectural parameters [22], leaf area index [23], or leaf biochemical constituents [24]. Additionally, in situ optical systems—mainly RGB cameras and multispectral and hyperspectral sensors—were also developed to retrieve different phenotypic traits with great detail. At the canopy scale, Diago *et al.* [25] developed an automatic method to classify horizontal RGB images of vines predicting plant leaf area and fruit load and also to estimate indices such as NDVI (normalized difference vegetation index) and water stress [26]. At the leaf scale, different empirical approaches were proposed, relating spectral indices with traits associated with plant water status [27,28] or biotic stress [29]. More recently, computer vision techniques have been successfully applied to detect disease symptoms on vine leaves [30,31].

High-throughput methods were also used to access parameters of responses to water shortage [32] or to describe grape bunch architecture. A striking example is the characterization of more than 1,500 genotypes to identify QTLs for bunch architecture using a 3-dimensional (3D) scanner [33].

The use of LiDAR (light imaging detection and ranging) optical devices has progressively gained attraction in close-range phenotyping during the last decade [*34*]. Active LiDAR-based sensors provide an explicit 3D description of the canopy observed. This technology is highly relevant in the case of vineyards where an accurate description of the actual 3D architecture is necessary to estimate light interception and other downstream eco-physiological processes such as canopy-level photosynthesis or evapotranspiration [35]. The work conducted by Arnó *et al.* [*36*] was one of the first studies to use a ground LiDAR-based system to estimate architectural traits in vineyards. In such a study, different canopy architectural indicators obtained from LiDAR point clouds such as height, cross-sectional area, canopy volume, and tree area index were used to derive a leaf area index with satisfactory results. Later studies have explored as well the use of LiDAR sensors to estimate plant architectural traits, such as leaf inclination or orientation [37], to provide a sensor-based assessment of winter pruning weight [3,9] or trunk volumes [38].

The LiDAR technology has been already applied to describe genotype–phenotype relationships for canopy height response to temperature in wheat [39], to identify QTLs explaining canopy height in maize [40], and more recently for apple tree architecture [41]. However, the use of LiDAR sensors to describe the variability of plant vigor associated with genetic factors has never been exploited for the grapevine.

The main goal of the present study was to evaluate the performance of phenotypic traits estimated from ground LiDAR sensors data to unravel phenotype–genotype associations in a biparental progeny. We estimated canopy volume in summer and wood volume in winter from LiDAR-based measurements. The estimation of winter wood volume from LiDAR sensors was compared with destructive measurements of pruning weight. Canopy volume at véraison estimated from LiDAR sensors was compared against estimates of exposed leaf area (ELA), derived from RGB images. We employed a SPAD-502 device to assess how genetic variations in leaf chlorophyll content could elucidate the growth potential of the studied genotypes, knowing the linear correlations between chlorophyll concentrations in the leaves, SPAD-502 measurements, and vine biomass production [42],

Quantitative trait loci (QTL) detection was carried out to identify genomic regions associated with the genetic variability of the destructive—pruning weight—and nondestructive observations of canopy and wood volume, ELA, and leaf chlorophyll content and, then, assess the ability of each variable to detect genetic factors associated to plant vigor.

## Material and Methods

**Fig. 1.**
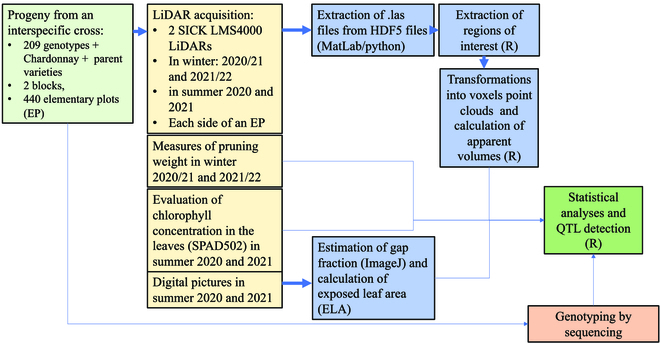
Overview of the experimental and technical design of the study.

The experimental and technical design of the study is presented in Fig. 1.

### Plant material and experimental design

Progeny from a cross between 2 grapevine interspecific hybrids, IJ119 (FRA038_PRO_HYB50001Col119) and Divona (VIVC 26503), was cultivated in the INRAE vineyard in Colmar, France. Both parents carry resistance genes to downy and powdery mildew.

Vines were grafted on the Selektion Oppenheim 4 (SO 4) rootstock (VIVC 11473) in 2015, planted in the field in 2016, and trained with a double Guyot system on a vertical shoot positioning system at a planting density of 4,200 plants per ha. Plants were not irrigated. All the vines were trellised and trimmed on the same date to control the whole canopy architecture.

The plants were distributed in 11 rows, with 1.7-m spacing between rows and 1.4 m between plants. A set of 209 offspring of the IJ119 × Divona progeny, the parents, and the control variety *Vitis vinifera* cv. Chardonnay were planted according to a randomized complete 2-block design. Each genotype was represented by one elementary plot of 3 plants in each block. The control variety, Chardonnay, was represented by 6 elementary plots of 3 plants per block. Each block included finally 220 elementary plots (209 genotypes from the progeny, 6 Chardonnay, one for each parent, and 3 as duplicates of 3 genotypes to avoid empty plots in the experiment, and not used in the study).

The soil, deeper than 90 cm, is classified by the World Reference Base for soil resources (https://www.fao.org/3/i3794en/I3794en.pdf) as “Bathyfluvic Calcaric Cambisol (Loamic)” with a loamy texture (>67%).

### Ground-truth data winter pruning weight

In winter, after leaf (BBCH 97) and before the beginning of bud swelling (BBCH 01) [43], we measured the fresh weight of pruning wood per elementary plot in the field. This was done on 2021 February 18, for both blocks 1 and 2, and only on block 1 on 2022 February 9. Block 2 did not receive any phytosanitary protection during the 2021 growing season to evaluate the resistance to diseases in the field. As a result, susceptible genotypes were severely affected, and measuring their pruning weight was meaningless. For the statistical analysis and QTL detection, only the genotypes with at least 2 adult productive plants in each of the 2 blocks were considered. The results are expressed as pruning fresh weight per plant (g.plant^−1^).

### RGB image-based retrieval of the ELA

**Fig. 2.**
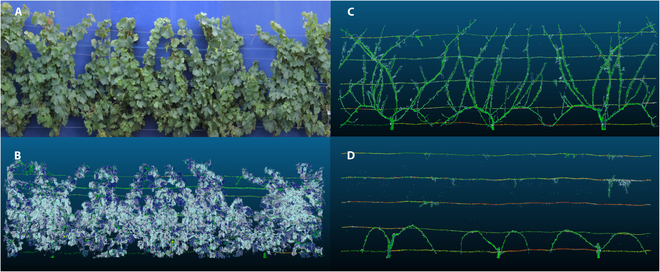
Images and point clouds for an elementary plot of Chardonnay. (A) Assembly of 3 photos taken on 2020 August 4. (B) Point cloud reconstructed from LiDAR sensors on 2020 August 5 (véraison). (C) Point cloud before pruning (2021 February 9). (D) Point cloud after pruning (2021 March 17).

The method used was already described [*13*]. In brief, digital images were taken manually by a pedestrian with a Canon 700D camera fitted with a Sigma 12-mm focal lens using a blue curtain as a background. In 2019, only the central plant of each elementary plot in block 1 was photographed. In 2020 and 2021, all the plants in block 1 were photographed. An ImageJ script was then used to calculate the ratio between “green” and “non-green” pixels for each picture. The final score was the percentage of foliage covering the area analyzed (FC). Figure 2A shows an example of images taken on a 3-plant plot of Chardonnay. An elementary plot may include some vegetative parts of the neighboring plants. This is a limitation of the study, which can induce some lack of accuracy of the results for all the methods, except for manual weighing of the pruning wood.

ELA, expressed in m^2^ per plant, was then calculated according to Carbonneau [*44*] as *ELA* = *D* × *FC* × *S*, where *S* is a constant calculated with the parameters of canopy geometry. *S* = 2.05 with our training system (width = 0.3 m, *H* = 1.40 , *S* = *W* + 1.25*H* for full sun). *D* is the distance between plants (1.40 m), and *FC* is the estimate for foliage coverage. Images were taken on 2019 August 2, 2020 August 4, and 2021 July 22, around véraison time (BBCH 85).

### Phenotyping apparent canopy and winter wood volume based on LiDAR point clouds

#### LiDAR data acquisition

**Fig. 3.**
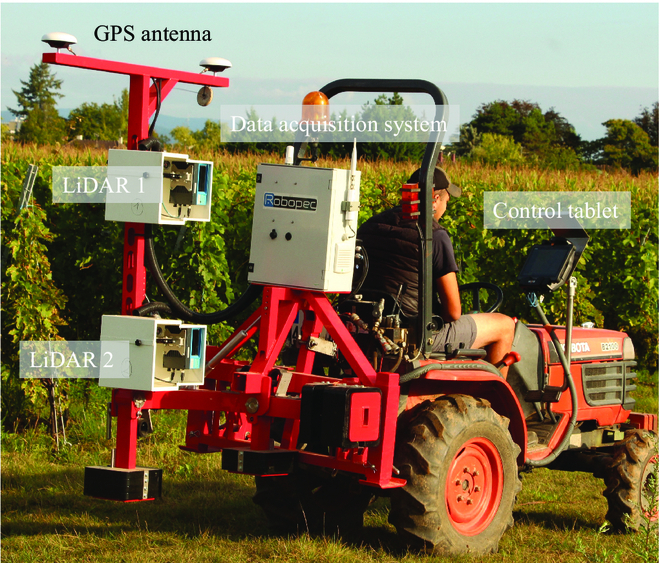
Overview of the system carrying the LiDAR sensors. The vertical distance between the LiDAR sensors was 0.70 m (RGB images for this study were obtained manually).

Point clouds of grapevine plants were obtained using the LiDAR technology. Two SICK LMS4000 LiDAR sensors were installed on a frame mounted on a tractor (Fig. 3). The vertical distance between the 2 LiDAR sensors was 0.7 m to capture the full canopy. The SICK LMS4000 LiDAR can retrieve information from 0.7 to 3 m and has an angular scanning range of 70° and a scanning frequency of 600 Hz. The angular resolution is 0.0833°. The tractor operated at a speed of about 2 km.h^−1^. The start and stop of LiDAR acquisitions were automatically set at the beginning and the end of each of the 440 elementary plots using the positions recorded by the Real Time Kinematik (RTK) Global Positioning System (GPS). Data were obtained for the 2 sides of each row. To limit the effects of neighboring plants, LiDAR data acquisition was triggered only 25 cm after the GPS signal of entering an elementary plot.

Several campaigns were performed: on 2020 August 5, around véraison (BBCH85) (block 1 only), on 2021 February 9 (before pruning), on 2021 March 17 (after pruning), around véraison (BBCH 85) in 2021 (August 30), and before (February 8) and after (March 8) pruning in 2022. An example of point clouds collected from LiDAR sensors in summer and winter (before and after pruning) is given in Fig. 2B to D.

#### Computing apparent volumes from 3D point clouds

Point clouds were generated from the GPS coordinates, the known distance between the GPS antenna and the 2 LiDAR sensors, and the sensor–target distance measured by the LiDAR, all contained in an HDF5 file for each elementary plot. Point clouds were then converted to a “.las” format and visualized with the CloudCompare software version 2.11.1 (www.cloudcompare.org) when necessary.

**Fig. 4.**
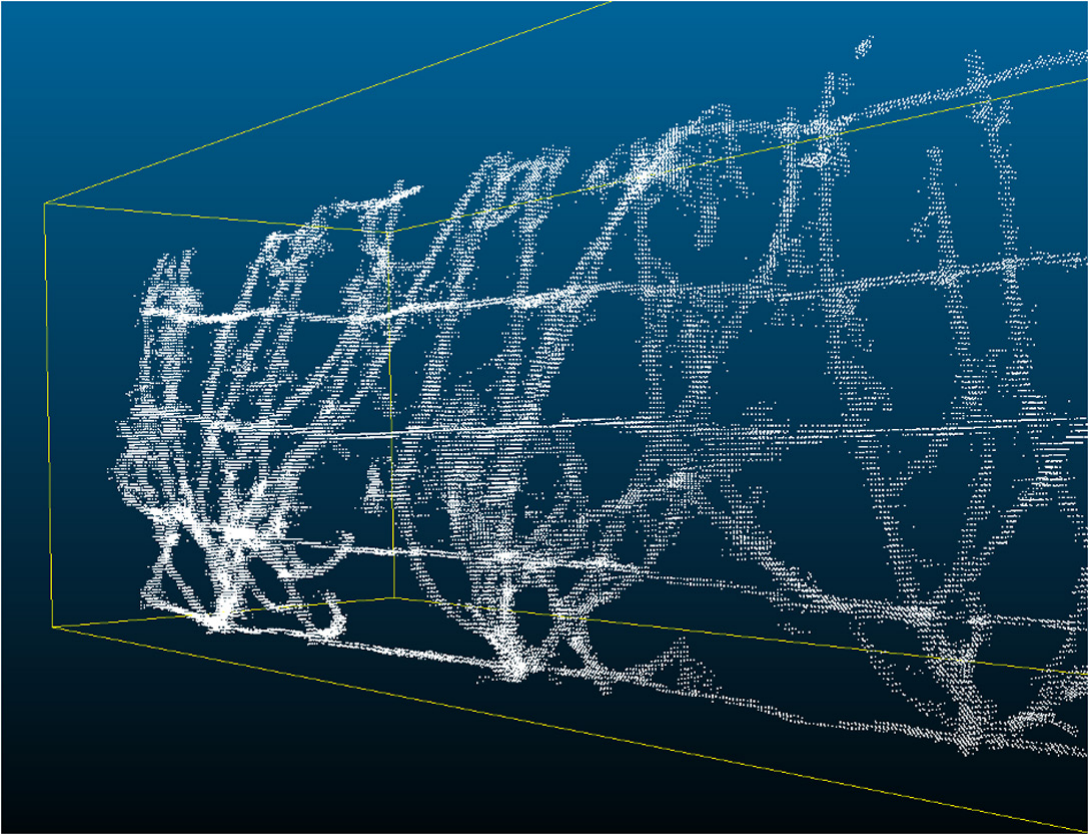
Example of a voxelized point cloud. Elementary plot 32305, genotype 1424S, bottom LiDAR sensor, north side of the row, before pruning. 2020/21 winter.

LiDAR point clouds were then processed to remove the soil and background impacts as well as the overlapping area covered by the 2 LiDAR instruments. The 3D point cloud scenes were divided either into 10-mm voxels for canopy data and 5 mm for pruning wood data. This size was chosen to minimize the volumes of the files while keeping a resolution adapted to the size of the organs to characterize (leaves and shoots). The voxels containing at least one beam impact were then classified as “vine,” whereas those voxels not containing any impact were considered empty. The classified voxel scenes were then split into 3 equal zones corresponding, respectively, to the 3 vines present in each elementary plot (example in Fig. 4).

Only elementary plots with at least 2 productive adult plants were kept for statistical analyses. Four values for the number of voxels classified as “vine” were available per plant: for the upper and the lower LiDAR sensor, and each side of the plant (north and south). Values for the upper and lower LiDAR sensor were added to obtain a single value for each side of the row.

The apparent volume for each plant for each side of the row was then computed as:

$$V_{i}=N_{v,i}*s$$

where *V_i_* is the apparent volume for plant *i* (expressed in L.plant^−1^), *N*_v,*i*_ is the number of voxels classified as “vine” in the area occupied by plant *i*, and *s* is the voxel size, in L. Volumes were not calculated by plot because some elementary plots did not contain 3 adult plants. In the LiDAR acquisitions taken at véraison, the apparent volume corresponds to the total canopy volume (including leaves and woody parts).

The objective was to compare pruning weights to data obtained from LiDAR sensors. We consequently sought to estimate, using the LiDAR sensors, the volume of wood removed from the plants. As suggested [3,45], the pruning wood volume was calculated as the difference between the whole plant volume retrieved from the acquisitions in winter before pruning minus the plant volume retrieved from the acquisitions after pruning. Only some small portions of wires visible after pruning were masked by the shoots before pruning (Fig. 2C and D). All the volumes calculated following this approach are probably slightly overestimating the actual canopy volumes as a voxel is considered full when containing a single impact. Moreover, the contribution of metallic wires to LiDAR impacts was considered negligible in summer (Fig. 2B) and neutralized by the subtraction approach used for winter data. For these reasons, we prefer to use the term “apparent volume” to name LiDAR-derived volumes. Biases cannot be excluded. For example, wires are more visible in low-vigor genotypes, leading to an overestimation of apparent canopy volume. Such a bias would reduce genetic variability and lower the power of QTL detection. Anyhow, we hypothesized that the apparent volumes realistically describe the phenotypic differences.

### Sensor-based measurement of the total leaf chlorophyll content

The total chlorophyll content of the leaves was assessed with a Konica-Minolta SPAD-502 Chlorophyll Meter (Konica-Minolta Inc., Tokyo, Japan). Three consecutive sets (one set by plant) of 5 measures were taken on 5 different leaves midway up the north face of the canopy. The 3 means of the 5 measures per plant were used for statistical analysis. Measures were performed on 2020 June 4 (BBCH65) and 2021 August 25 (BBCH85).

### Statistical analyses and heritabilities

Statistical analyses were performed with R version 4.1.0 [46]. Heritabilities of the means can be defined as *H*^2^ = σ_g_^2^/(σ_g_^2^ + σ_e_^2^), where σ_g_^2^ is the genetic variance and σ_e_^2^ is the environmental variance. When data were obtained on 2 blocks (pruning weight and LiDAR data in February and March 2021), σ_g_^2^ and σ_e_^2^ were directly extracted from analyses of variance (ANOVAs). However, data from block 2 were not available in 2021 because this block was not protected against foliar diseases. To allow meaningful comparisons among all the datasets, we chose to calculate the heritabilities only on block 1 using the 6 control plots of Chardonnay planted across this block. If σ_t_^2^ is the variance observed over all the genotypes from the progeny in block 1 and σ_e_^2^ is the variance among the 6 plots of Chardonnay, we calculated broad-sense heritabilities as *H*^2^ = (σ_t_^2^ − σ_e_^2^)/σ_t_^2^. If applicable, the best linear unbiased predictors were extracted from ANOVAs during the process.

### Genotyping and genetic map construction

Genomic DNA was extracted from young expanding leaves of 249 genotypes grown in the greenhouse using the Qiagen DNeasy 96 Plant Kit (Qiagen S.A., Courtaboeuf, France) as described by the supplier. Following quality control, DNA samples were analyzed with genotyping by sequencing (GBS), a method used to unravel single-nucleotide polymorphisms (SNPs) [47]. DNA from each genotype was digested by the ApeKI restriction enzyme to obtain DNA fragments of 100 base pairs (bp). After the ligation of polymerase chain reaction adapters and barcodes, DNA fragments from 96 genotypes were pooled together to form GBS banks that were sequenced on an Illumina HiSeq4000 platform (paired-end, 2 × 100 bp).

The reads were aligned on the *V. vinifera* “PN40024.v4” reference genome [48] using BWA-MEM (Burrows-Wheeler Aligner) [49].

SNP calling was performed using the gstacks command of Stacks v2 pipeline [50]. The output file in Joinmap format was filtered to only keep the most informative and reliable markers. SNPs with more than 10% missing data, with non-Mendelian segregation (χ^2^ test at *P* = 0.05) or not consistent with the genotype of the parents, were discarded.

Parental and average genetic maps were constructed using Lep-Map3 [51]. The ParentCall2 module of Lep-MAP3 was used to call parental genotypes, the SeparateChromosomes2 module was used to split the markers into 19 linkage groups (at LOD 36), and the OrderMarkers2 module was used to order the markers within each linkage group using 30 iterations per group, and finally computing genetic distances. The phased output data were converted into R/qtl format (4way-cross) for R software [52].

Before QTL detection, redundant markers were removed using the findDupMarkers command in R/qtl.

### QTL detection

QTL detection was performed on the consensus map with the R/qtl software [52] using the multiple imputation method (“draws” = 64) and the one-dimension scan command scanone. LOD (logarithm of odds; that evaluates the likelihood of the presence of a QTL) significances were ensured with permutation tests (1,000 permutations). QTL models that combine the additive effects of relevant loci, including interactions when significant, were constructed step by step after the refinement of the QTL position (refineqtl) and the search for supplementary QTLs (addqtl). The LOD score and the percentage of variance explained by a QTL in a QTL model were assessed with ANOVAs using type III sums of squares (fitqtl). The fitqtl command also provides the overall LOD value and the percentage of variance explained by the complete QTL model. The confidence intervals were calculated as Bayesian credible intervals (bayesesint) with a probability of coverage of 0.95. As the position of markers is included in the names of the markers, using the “expand to marker = true” option allowed direct access to the physical positions of the confidence intervals in the PN40024.v4 reference genome [53]. The LinkageMapView package [54] was used for drawing QTL positions on the consensus genetic map.

## Results

### Validation of LiDAR-derived canopy and wood volumes to assess vine growth

**Fig. 5.**
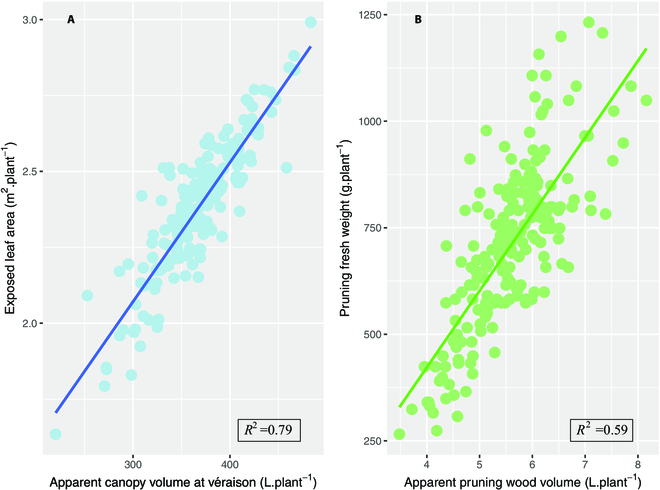
Relationships between apparent volumes calculated with LiDAR data and (A) exposed leaf area and (B) pruning fresh weight for the 2020/2021 growing season.

**Fig. 6.**
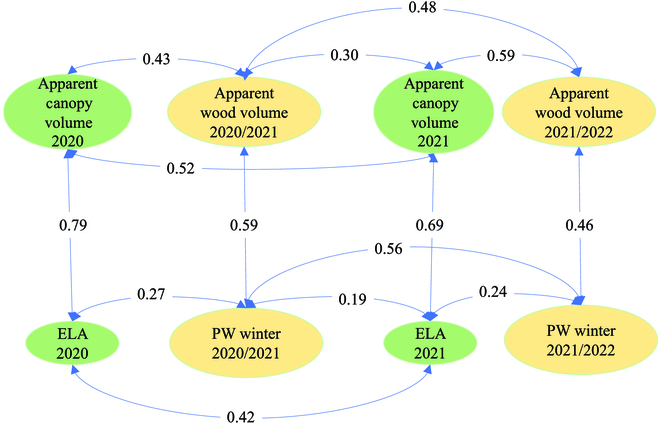
Diagram showing the coefficients of determination *R*^2^ between relevant variables. All the relationships were statistically significant at least at *P* < 0.001. PW, pruning weight; ELA, exposed leaf area calculated from RGB images. Green, variables for the canopy in summer; yellow, variables for the dormant tissues in winter.

The relationships between LiDAR-derived canopy volumes against ELA and pruning weight for the 2020/2021 growing season are presented in Fig. 5. The apparent canopy volume at véraison is strongly correlated with the ELA measured from digital photographs (*R*^2^ = 0.79), which is considered the reference methodology to measure this trait in the field. In the 2021/2022 growing season, the correlation is also high (*R*^2^ = 0.69; Fig. 6) but analyses of covariance show that the mathematical relationships between apparent canopy volumes and ELA are not the same for the 2 growing seasons (Table S1). The correlation between years was better with LiDAR data than with digital images (0.52 versus 0.42; Fig. 6), which suggests that our method of image analysis is less reproducible, presumably due to uncontrolled lighting conditions.

Similarly, apparent pruning wood volume derived from LiDAR point clouds is positively correlated with destructive measurements of pruning fresh weight (*R*^2^ = 0.59 and 0.46 in 2020/2021 and 2021/2022, respectively; Figs. 5B and 6). However, the relationship between the apparent wood volume estimated with LiDAR data and the weight tends to be weaker as the values increase (Fig. 5B). The linear model describing this relationship is different in the years 2020 and 2021 (Table S2). Figure 6 also shows that the correlations between apparent canopy and wood volume, both derived from LiDAR, are stronger (0.43 < *R*^2^ < 0.59) that the ones observed between ELA and pruning weight (0.24 < *R*^2^ < 0.27). We have no convincing explanation for the better correlations between pruning weights between the 2 seasons (*R*^2^ = 0.56) than for apparent wood volumes (*R*^2^ = 0.48). A weak but significant opposition between the chlorophyll content and the ELA was observed in 2020 (Table S3).

### Heritability of direct and sensor-based measurements of vine growth and chlorophyll content

**Fig. 7.**
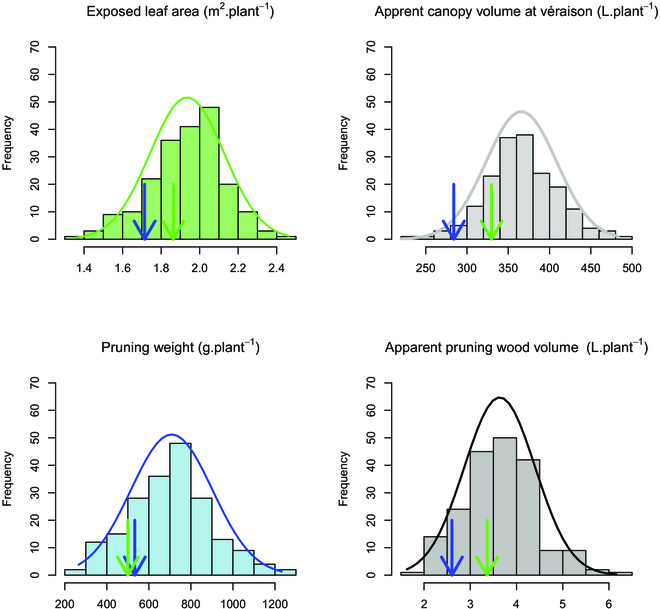
Segregations observed for 4 traits for the 2020/2021 growing season. Blue arrow, IJ119; green arrow, Divona. All these segregations did not diverge significantly (*P* > 0.05) from a normal distribution according to a Shapiro–Wilk test (Table S4).

All the traits, except ELA 2019 and 2021, did satisfy the criteria of normality according to a Shapiro–Wilk test (Fig. 7 and Table S4). The statistics for all the ground-truth and LiDAR-based measurements (ELA, pruning weight, canopy, and wood apparent volumes) for the Chardonnay cultivar in block 1, the parent genotypes, and the progeny are presented in Table 1.

**Table 1.** Descriptive statistics, heritabilities of the traits, and differences between the 2 parents

Trait	Vintage	Progeny					Chardonnay		*H* ^2 a^	Divona	IJ119	*P* value^b^
		n	Min.	Mean	Max.	Var.	Mean	Var.		mean	Mean	
Exposed leaf area (m^2.^plant^−1^)	2019	177	0.99	1.96	2.59	0.12	1.70	0.02	0.84	2.56	1.47	NA
	2020	204	1.35	1.93	2.46	0.04	1.94	0.02	0.44	1.86	1.71	0.51
	2021	206	1.57	2.14	2.57	0.05	2.04	0.04	0.16	2.33	1.84	<0.001
Chlorophyll content (unit less)	2020	204	242.0	316.8	403.8	854.5	298.4	268.3	0.69	281.7	293.0	0.60
	2021	208	233.9	320.7	375.2	713.1	327.3	51.3	0.93	280.8	268.3	0.62
Apparent canopy volume at véraison (L.plant^−1^)	2020	184	220.2	365.6	483.4	1,850.8	345.8	393.3	0.79	329.7	284.0	0.03
	2021	172	214.4	305.4	390.7	1,334.1	269.9	455.9	0.66	305.9	298.3	0.85
Apparent pruning wood volume (L.plant^−1^)	2020	197	1.54	3.59	5.75	0.727	3.04	0.122	0.83	3.36	2.60	0.001
	2021	199	2.15	4.96	8.15	1.13	3.51	0.113	0.79	3.37	2.68	NA
Pruning fresh weight (g.plant^−1^)	2020	197	200	709	1,466	44,316	700	29,777	0.33	500	533	0.73
	2021	201	240	677	1,390	41,879	523	4,426	0.89	341	345	NA

^a^ Heritability. ^b^For the difference between the 2 parents. *P < 0.05, **P < 0.01, ***P < 0.001. NA, data on one block only.

The parents were significantly different for at least 1 year for all traits, except for chlorophyll content. Divona was more vigorous than IJ119 for canopy parameters.

Considering the 2 seasons, LiDAR-derived traits on plant growth, apparent wood volume, and apparent canopy volume presented high and stable heritabilities (Table 1). Heritability was above 0.79 for the apparent pruning wood volume, and above 0.66 for the total canopy volume at véraison. The ELA presented a high heritability only in 2019 (0.84) but was substantially lower in the years 2020 and 2021 (0.44 and 0.16, respectively). Similarly, the heritability of the actual pruning weight was quite variable depending on the year: 0.33 in 2020 and 0.89 in 2021.

The heritability of leaf chlorophyll content was also high (0.69 in 2020 and 0.93 in 2021), comparable, in terms of genetic variance explained, to the apparent volume of pruning wood.

### Genetic maps and detection of QTLs for canopy volume and pruning wood

Genetic information from 249 genotypes of the population was used for the construction of 2 parental maps and one consensus map, which all display 19 linkage groups and a high marker density of 0.1 cM between markers, on average. The consensus map, with a total length of 1,017.3 cM, contains 28,274 SNP. The female map has 19,935 SNPs with a total genetic length of 1,110.1 cM, and the male map has 20,134 SNPs covering 1,177 cM. Genetic and physical distances of markers in the maps are highly correlated (*r* > 0.97, Spearman correlation test). After removing redundant markers, the genetic files used for QTL detection contained 1,582, 1,803, and 4,473 SNPs for the female, male, and consensus map, respectively. Detailed information is provided in Table S5 and Fig. S1.

**Fig. 8.**
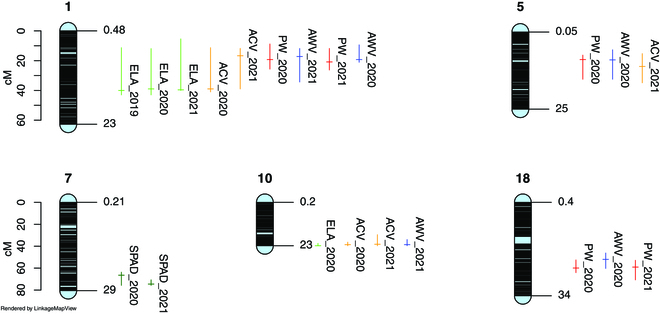
Positions of the main QTL detected on the consensus map. Only QTLs detected for at least 2 years are presented. Vertical lines, Bayesian credible interval with 0.95 probability of coverage; horizontal line, position of the LOD peak. ELA, exposed leaf area; PW, pruning weight; SPAD, chlorophyll content measured with a Konica-Minolta SPAD 502; ACV, apparent canopy volume; AWV, apparent wood volume.

QTLs detected for canopy features were highly significant (Table 2 and Fig. 8). For the ELA estimated on digital images, only a QTL on chromosome 1 was significant for the 3 growing seasons. QTLs on chromosomes 3, 10, and 19 were detected for one season only. With LiDAR data, QTLs for apparent canopy volume on chromosomes 1 and 10 were detected in both 2020 and 2021. They had higher LOD scores than QTLs for ELA, and the complete QTL models (Table 4) explained approximately 28% and 44% of the total variance in 2020 and 2021, respectively, which was higher than for ELA.

**Table 2.** QTLs detected for the canopy in the consensus map

Variable	Vintage	Chrom.	Marker inf.	Borne inf (cM)	Borne inf (bp)	Marker max.	Pos. max (cM)	Pos. max (bp)	Marker sup	Borne sup (cM)	Borne sup (bp)	LOD threshold at *P* = 0.05	LOD Max	%Var
Exposed leaf area	2019	1	chr1_3090257	11.3	3090257	chr1_9317790	40.0	9317790	chr1_10780610	43.0	10780610	4.50	5.09	12.4
	2020	1	chr1_3080452	11.9	3080452	chr1_9345396	39.0	9345396	chr1_10780610	43.0	10780610	4.27	5.53	10.4
		10	chr10_18027354	37.4	18027354	chr10_23741952	39.6	23741952	chr10_23582004	39.6	23582004	4.27	6.95	13.3
	2021	1	chr1_1513051	5.4	1513051	chr1_3080452	11.8	3080452	chr1_7439817	32.3	7439817	4.32	6.71	11.9
		3	chr3_144244	1.2	144244	chr3_3305060	18.3	3305060	chr3_4629328	30.5	4629328	4.32	4.77	8.3
		19	chr19_18046877	36.0	18046877	chr19_22101414	42.2	22101414	chr19_23554673	46.2	23554673	4.32	4.63	8.0
Apparent canopy volume at véraison	2020	1	chr1_3090257	11.3	3090257	chr1_9345396	39.0	9345396	chr1_9705116	41.0	9705116	4.39	7.93	16.29
		10	chr10_17185371	36.3	17185371	chr10_22825196	38.6	22825196	chr10_23582004	39.6	23582004	4.39	6.68	13.5
	2021	1	chr1_3080452	11.8	3080452	chr1_3740718	16.7	3740718	chr1_9217371	39.0	9217371	4.45	9.30	15.7
		10	chr10_10672133	29.5	10672133	chr10_22058347	38.2	22058347	chr10_23582004	39.6	23582004	4.45	6.24	10.1
		9	chr9_3328455	1.2	3328455	chr9_6306135	10.2	6306135	chr9_9235678	15.1	9235678	4.45	6.01	9.7
		5	chr5_4706286	15.3	4706286	chr5_6248776	23.9	6248776	chr5_11731166	34.9	11731166	4.45	5.10	8.1
Chlorophyll content	2020	7	chr7_23198477	63.7	23198477	chr7_23715663	66.5	23715663	chr7_27803890	75.7	27803890	4.26	7.93	14.2
		7	chr7_228009	0.4	228009	chr7_1603588	7.6	1603588	chr7_9546705	34.1	9546705	4.26	4.50	7.7
		13	chr13_669746	5.4	669746	chr13_2875318	10.6	2875318	chr13_6773780	26.1	6773780	4.26	5.25	9.1
	2021	7	chr7_2533757	70.7	25337572	chr7_26876058	74.5	26876058	chr7_27803890	75.7	27803890	4.50	5.06	10.6

QTLs for the chlorophyll content on chromosomes 7 and 13 did not colocalize with QTLs for the canopy volume, suggesting that the genetic factors controlling the chlorophyll content of the leaves are independent of genetic factors controlling growth capabilities in our context.

In 2021 (2020 growing season), apparent wood volumes, estimated with LiDAR data, and actual pruning wood weights were under the control of common QTLs on chromosomes 1, 5, and 18 (Table 3 and Fig. 8). Additional QTLs for apparent wood volume were detected on chromosomes 3 and 19 and an additional QTL for pruning weight on chromosome 8 (Table 3 and Fig. 8).The overall variance explained by these QTLs reached 48% for the apparent wood volume and 37% for the pruning weight, which is in both cases very satisfactory (Table 4).

**Table 3.** QTLs detected during winter in the consensus map

Variable	Vintage	Chrom.	Marker inf.	Borne inf (cM)	Borne inf (bp)	Marker max	Position Max (cM)	Position Max (bp)	Marker sup	Borne sup (cM)	Borne sup (bp)	LOD threshold at *P* = 0.05	LOD Max	%Var
Apparent pruning wood volume	2020	1	chr1_2543463	9.4	2543463	chr1_4109564	19.3	4109564	chr1_4313647	21.1	4313647	4.3	12.3	17.4
		3	chr3_1124937	5	1124937	chr3_1766835	8.8	1766835	chr3_2612920	15.3	2612920	4.3	6.1	8
		5	chr5_4445752	12.7	4445752	chr5_5617437	19.5	5617437	chr5_9669620	32.5	9669620	4.3	4.8	6.2
		18	chr18_10151265	46.2	10151265	chr18_11270002	52	11270002	chr18_13475622	60.3	13475622	4.3	6.1	8.1
		19	chr19_8878385	25.1	8878385	chr19_9478850	28.5	9478850	chr19_9766230	30.1	9766230	4.3	9	12.2
	2021	1	chr1_3099564	11.85	3099564	chr1_3783694	17.27	3783694	chr1_8029908	34.34	8029908	4.4	5.31	9.3
		9	chr9_3076366	0.6	3076366	chr9_3659812	2.2	3659812	chr9_6058593	10.04	6058593	4.4	5.11	8.9
		10	chr10_13160924	33.93	13160924	chr10_22762697	38.59	22762697	chr10_23601946	39.56	23601946	4.4	6.09	10.8
Pruning weight	2020	1	chr1_2479357	8.8	2479357	chr1_4109564	19.3	4109564	chr1_5293722	25.7	5293722	4.33	7.47	12
		5	chr5_5088536	16.3	5088536	chr5_5571412	19.3	5571412	chr5_9669620	32.5	9669620	4.33	6.48	10.3
		8	chr8_2739496	0	2739496	chr8_13490375	20.9	3490375	chr8_14001221	25.3	14001221	4.33	5.59	8.8
		18	chr18_11386757	52.5	11386757	chr18_13155251	59.9	13155251	chr18_18446488	64.1	18446488	4.33	6.31	10
	2021	1	chr1_3080452	11.84	3080452	chr1_4341347	20.88	4341347	chr1_5506983	26.3	5506983	4.39	4.81	8.3
		9	chr9_3091124	0.4	3091124	chr9_3328455	1.21	3328455	chr9_5448053	7.43	5448053	4.39	5.09	8.8
		18	chr18_11454398	52.85	11454398	chr18_12942084	59.08	12942084	chr18_25595240	70.72	25595240	4.39	6.27	9.9

**Table 4.** Summary of QTL results

Season	Vintage	Trait	Chromosomes	Overall LOD	%Var
Summer	2020	Exposed leaf area	1,10	10.91	21.8
		Apparent canopy volume at véraison	1,10	12.3	27.8
		Chlorophyll content	7,7,13	14.31	27.6
	2021	Exposed leaf area	1,3,19	14.04	27.0
		Apparent canopy volume at véraison	1,10,9,5	21.86	44.3
		Chlorophyll content	7	5.06	10.6
Winter	2020	Pruning weight	1,5,8,18	19.79	37.0
		Apparent pruning wood volume	1,3,5,18,19	27.66	47.6
	2021	Pruning weight	1,9,18	14.75	28.8
		Apparent pruning wood volume	1,9,10	14.54	28.6

In 2022 (2021 growing season), the total percentages explained by the QTLs, for both apparent wood volume and pruning weight, were lower than in 2021, around 29%. Two QTLs for pruning weight, on chromosomes 1 and 18, were already detected in 2021, but this was only the case for the QTL on chromosome 1 for the apparent wood volume.

Regardless of the methods used, only the QTL on chromosome 1 is stable, detected over 2 growing seasons, both in winter and in summer.

Considering the objective of the study, the heritabilities of the traits and the detection of QTLs show that the use of LiDAR technology to estimate the volume of the grapevine canopy in summer and the volume of dormant tissue volume in winter is suitable for genetic studies and is more powerful than previously used methods.

## Discussion

### Reliability of LiDAR-derived volume indicators to identify genetic differences in vine growth

The reliability and relevance of LiDAR-derived apparent volumes for characterizing vine vigor will be ultimately validated if we can show that these variables are useful for analyzing variation in agronomic traits such as total biomass production, yield level, or whole canopy transpiration. At this stage, validation is based on correlations with other variables, heritabilities, and, finally, power for QTL detection.

First, we showed significant correlations, although sometimes moderate, of LiDAR-derived apparent volumes with ground-truth indicators such as ELA or pruning weight (Fig. 6). More importantly, heritabilities calculated with LiDAR-derived indicators were more stable than those calculated with ground-truth variables. As shown in Table 1, the apparent pruning wood volume, as estimated with LiDAR sensors, shows a consistently high heritability (*H*^2^ ≈ 0.8) in both the 2020 and 2021 years. Similarly, the heritabilities of the apparent canopy volume at véraison are relatively high and consistent across years (*H*^2^ = 0.79 in 2020, *H*^2^ = 0.66 in 2021). These results show that both LiDAR-derived indicators are more relevant for characterizing genetic differences in vine growth than ELA and destructive pruning weight for which heritability was low (below 0.4) in at least one of the seasons. Although leaf chlorophyll content also has high heritability in both seasons (*H*^2^ = 0.69 in 2020, *H*^2^ = 0.93 in 2021), it is poorly correlated with ELA (Table S1). This suggests that chlorophyll content, as estimated with the Konica-Minolta SPAD 502 chlorophyll meter, is probably the expression of a different functional trait (see the next section).

Two factors may explain the greater reliability of the apparent canopy volume compared to ELA for characterizing genetic differences in growth. The first one is that the LiDAR system provides a proxy to the actual canopy volume through the identification of 3D voxels containing leaves and stems, whereas the ELA from RGB images is based only on an estimation of row porosity. Of course, canopy sampling by LiDAR beams may be subjected to occlusions and overlap between plant organs [55] that may bias the retrieval of the true canopy volume. But, in our experimental plan, the LiDAR acquisitions were conducted on both sides of the rows, which should have minimized this effect. In addition, because the manual process is time consuming, the porosity estimates from the digital images were only collected from one side of the rows. This made the estimates less accurate. The manual method for image acquisition and processing we used was quite simple but time consuming. We did not use the RGB cameras embedded in the system (Fig. 3) because we could not control the illumination conditions and remove the background, which is still challenging [56]. We plan to control the scenes with flashes to compare the LiDAR data with high-throughput RGB imagery.

The second factor is that the segmentation of RGB images can be influenced by lightning conditions, which can vary between the beginning and end of hundreds of shots. Therefore, the thresholds used during the segmentation process may not provide the same results depending on the skylight. This could explain why the heritability of ELA was low (0.16) in 2021, mainly due to a higher variance of Chardonnay data than in previous seasons. In contrast, LiDAR is an active device not very sensitive to natural lighting conditions. In addition, LiDAR processing requires only minimal parametrization—the voxel size and the number of hits to classify a voxel as canopy—to compute the apparent volumes, ensuring the repeatability of measurements, across dates and years.

We cannot definitively conclude that LiDAR sensors are more relevant than images to characterize grapevine growth parameters. RGB images can also provide information on leaf area and yield [25] and, when using stereo vision software, can also generate 3D point clouds [57]. Images are of interest not only for the identification of objects as small as berries but also for information about the color of the canopy. Indeed, one of the goals of canopy characterization is to estimate not only leaf area but also its global photosynthetic capacity, and LiDAR sensors alone cannot achieve this goal. Calculations of indices such as NDVI or GNDVI (green normalized difference vegetation index) with multispectral cameras and estimation of the water status of the plant by thermography [26] will add significance to estimates of light interception by the canopy.

For dormant tissues, the heritability of apparent pruning volume obtained with LiDAR was higher than that of pruning weight. The low heritability of pruning weight in 2020 (*H*^2^ = 0.33; Table 1), when compared to that of the apparent pruning wood volume (*H*^2^ = 0.83), could be partially explained by differences in dry matter content. The dry matter content of the shoots may indeed vary according to the variety, environmental conditions, and the level of reserves in the plants [58].

The correlations between LiDAR-estimated apparent wood volumes and pruning weights (*R*^2^ = 0.59 in 2020 and *R*^2^ = 0.46 in 2021) are lower than those reported by Siebers *et al.* [3] (*R*^2^ > 0.92), Tagarakis *et al.* [9] (*R*^2^ = 0.65, in 2010, *R*^2^ = 0.69 in 2011), or Moreno *et al.* [45] (*R*^2^ = 0.75). These authors, however, established the relationships only for one variety, and the data presented by Tagarakis et al. [9] do not allow us to conclude whether the slopes were identical for the 2 vintages studied. When studying 2 varieties, Sauvignon Blanc and Syrah over 2 vintages, Anastasiou et al. [59] concluded that the relationships between LiDAR-derived volumes and pruning weights were more accurate for Syrah than for Sauvignon blanc. The most appropriate method of data acquisition, as well as the accuracy of the relationships, was also different between the 2 vintages, without a clear explanation. The hypothesis that the linear equations between LiDAR-derived volume and pruning weight are genotype specific could explain the overall lower accuracy in our case. We can propose 2 hypotheses to be tested: (a) differences in wood density between varieties and (b) a different contribution of the main stems and the laterals to the pruning wood according to the variety.

Pruning wood estimates can also be obtained by digital images [60,61], but in both studies, the coefficients of correlation obtained after an automatic process were lower than with a manual process. Controlling the illumination as well as removing the background, either by taking images at night [61] or by using stereo vision [60], remains challenging. Our results indicate, anyhow, that dormant tissue volumes are better descriptors of the genetic differences in vigor than weights, which may be more influenced by the environment.

The apparent volumes extracted from LiDAR data in winter and in summer rely on relatively simple methods. We believe that the methodology we used is sufficient if the goal is to describe growth variability across a panel of genotypes in relative terms, but LiDAR analyses could still be improved using indices such as tree area index [36] or alpha-shape volumes [45,62]. To compute absolute volumes [63] or to derive plant leaf area, either physical methods based on the analysis of canopy transmittance from LiDAR beams [55] or empirical models [64] should be used.

Future studies should evaluate the suitability of more advanced LiDAR point cloud analysis methods for the segmentation of individual shoots on plants and the computation of shoot-scale traits such as shoot diameter or inclination angle, which can provide a deeper insight into the genetic determinants of grapevine vigor and plant architecture.

### Identifying QTL associated with vigor in a vine progeny using non-destructive canopy measurements

GBS is a now commonly used technology for unraveling the genotype–phenotype relationships for the grapevine. Our genetic parental maps have similar lengths and marker numbers, above 1,000, than recently published data [65–68]. The main difference is that we used in this study a consensus map calculated with LepMap3. This map finally enclosed more than 4,773 markers while staying compact (1,017.3 cM). Analyzing the origin of some specific features of our maps is not in the scope of this article.

The progeny used in this work proved very well adapted to seek the genetic determinants of grapevine vigor. Indeed, all the traits under study segregated in this progeny and we identified loci in the grapevine genome associated with these variations in both growing seasons: a QTL on chromosome 1, for all the traits, except chlorophyll content, an additional QTL on chromosome 10 for the apparent canopy volume, a QTL for pruning wood weight on chromosome 18, and a QTL for chlorophyll content on chromosome 7. Several other loci, on chromosomes 3, 5, 8, 9, 13, and 19, had less reproducible effects. These QTL should be confirmed with a third year of data, but we provide here preliminary insights into the genetic determinism of grapevine vigor.

We detected a QTL for the chlorophyll content of the leaves on chromosome 7. In the results of Bert et al. [69], who identified a reproducible QTL for SPAD measurements on chromosome 1, this locus is never mentioned. SPAD-502 measurements are well correlated to the level of nitrogen nutrition and growth parameters when considering a single variety [42]. However, while the relationship between SPAD-502 measurements and actual measurements of leaf chlorophyll concentration may be unique, the relationship between SPAD-502 measurements and leaf nitrogen concentrations is cultivar dependent [42]. The lack of colocalization of the QTL for SPAD-502 measurements with the other growth-related traits may indicate that leaf chlorophyll concentration, despite its variability, never limited growth capabilities, regardless of genotype. Indirectly, this also suggests that nitrogen nutrition was not a limiting factor in our study. Data acquisition with the Konica-Minolta SPAD-502 was performed manually, but the detection of genetic variation in chlorophyll concentrations and associated loci is a positive signal for the development of a high-throughput multispectral imaging system that could provide us with indices related to leaf photosynthetic activity.

For growth parameters, a QTL on chromosome 1 was found whatever the growing season, the method used, or the period of measurement (summer or winter). Differences in QTL detection between winter and summer data were expected: genetic variations of internode length [70] can induce variations in the number of leaves, and leaf area, for the same shoot length. Differences between growing seasons can also result from a limiting factor, like water availability, more or less revealing the genotypic susceptibility. A deeper analysis of the genes in the confidence intervals of the QTL will be conducted to propose candidate genes that could explain the genetic variations of the traits. A QTL for pruning weight, for one season, was already detected on chromosome 1 in a Riesling × Gewurztraminer progeny [71]. The confidence interval of this QTL (6,527,798 to 20,729,170 bp in the PN40024.v4 genome) overlaps with the confidence intervals of the QTL we detected for the canopy on the same chromosome (Table 2 and Fig. 1). QTLs related to plant growth were detected in progenies used as rootstocks [12,70], but comparisons with our results would not be relevant.

One of the main outcomes of our study is that LiDAR sensors are more powerful for deciphering the genetic determinism of grapevine growth parameters than reference methods. For the canopy, the LOD scores for apparent volumes were higher in both 2020 and 2021, but, more importantly, the total percentage of variance explained by the models varied between 21.8 and 27.0 % for ELA, in 2020 and 2021, respectively, whereas it reached 27.8% and 44.3% in 2020 and 2021, respectively, for apparent volumes calculated from point clouds (Table 4). The same conclusion can be drawn from the pruning wood QTL (Table 3), with up to 47.6% of the variance explained by LiDAR technology versus 37.0% for pruning weight in 2020.

An important question is whether monitoring vine vigor in winter is necessary to describe genetic variations in vine growth. Pruning weight has traditionally been used as an indicator because it is easy to measure. However, the most important traits to consider in genetic studies are those that describe the ability of the canopy to synthesize sugars and those that are related to the light environment and microclimate around the grapes. Describing the canopy in summer using traditional methods, including digital pictures, is time consuming and was rarely performed on collections of genetic variants [13]. With high-throughput methods such as automated LiDAR acquisition, the kinetics of canopy development between flowering and harvest are accessible. In addition to its interest for QTL detection, the dynamics of light interception from high-throughput methods could be introduced into biomass production models. Ultimately, integration into berry sugar concentration models may help to unravel the genetic origins of variations in berry sugar content. Meanwhile, the data obtained in this work will be integrated into the analysis of genetic variation in sugar accumulation observed in this progeny.

## Conclusion

Our main objective was to validate the use of LiDAR sensors for high-throughput phenotyping of grapevine growth-related traits. The LiDAR-derived apparent volumes at véraison—total canopy volume—and in winter—pruning wood volume—showed high and more stable heritabilities than ELAs measured from images and destructive measurements of the pruning weight. They were further validated by powerful QTL detection. These results highlight the reliability of LiDAR-derived traits for characterizing genetic differences in grapevine growth, which can be used instead of traditional low-throughput methods. Our study also opens new perspectives for high-throughput phenotyping of grapevines in the vineyard to describe not only genetic variation but also the effects of environmental conditions, training systems, or management techniques.

## References

[1] Carvalho LC, Goncalves EF, da Silva JM, Costa JM. Potential phenotyping methodologies to assess inter- and Intravarietal variability and to select grapevine genotypes tolerant to abiotic stress. Front Plant Sci. 2021;12:718202. 34764964 10.3389/fpls.2021.718202 PMC8575754

[2] Kicherer A, Herzog K, Bendel N, Klück HC, Backhaus A, Wieland M, Rose J, Klingbeil L, Läbe T, Hohl C, et al. Phenoliner: A new field phenotyping platform for grapevine research. Sensors. 2017;17(7):1625. 28708080 10.3390/s17071625 PMC5539483

[3] Siebers MH, Edwards E, Jimenez-Berni J, Thomas M, Salim M, Walker R. Fast Phenomics in vineyards: Development of GRover, the grapevine rover, and LiDAR for assessing grapevine traits in the field. Sensors. 2018;18(9):2924. 30177637 10.3390/s18092924 PMC6163379

[4] Kraus C, Pennington T, Herzog K, Fisher M, Voegele RT. Effects of canopy architecture and microclimate on grapevine health in two training systems. Vitis. 2018;57(2):53–60.

[5] Valdés-Gómez H, Gary C, Cartolaro P, Lolas-Caneo M, Calonnec A. Powdery mildew development is positively influenced by grapevine vegetative growth induced by different soil management strategies. Cop protection. 2011;30(9):1168–1177.

[6] Cocco A, Mercenaro L, Muscas E, Mura A, Nieddu G, Lentini A. Multiple effects of nitrogen fertilization on grape vegetative growth, berry quality and pest development in Mediterranean vineyards. Horticulturae. 2021;7(12):530.

[7] Munitz S, Schwartz A, Netzer Y. Effect of timing of irrigation initiation on vegetative growth, physiology and yield parameters in cabernet sauvignon grapevines. Aust J Grape Wine Res. 2020;26(3):220–232.

[8] Zufferey V, Murisier F, Vivin P, Blecher S, Lorenzini F, Spring JL, Viret O. Carbohydrate reserves in grapevine (*Vitis vinifera* L. ‘Chasselas’): The influence of the leaf to fruit ratio. Vitis. 2012;51(3):103–110.

[9] Tagarakis AC, Koundouras S, Fountas S, Gemtos T. Evaluation of the use of LIDAR laser scanner to map pruning wood in vineyards and its potential for management zones delineation. Precis Agric. 2018;19(2):334–347.

[10] Hugalde IP, Agüero CB, Barrios-Masias FH, Romero N, Viet Nguyen A, Riaz S, Piccoli P, McElrone AJ, Walker MA, Vila HF. Modeling vegetative vigour in grapevine: Unraveling underlying mechanisms. Heliyon. 2020;6(12):e05708. 33385078 10.1016/j.heliyon.2020.e05708 PMC7770548

[11] Li MM, Yan X, Guo Z, Jia N, Yuan J, Han B, Yin Y, Sun Y, Liu C, Zhao S. Rootstock influence on vegetative growth, yield, and fruit quality of 'Petit Verdot'. Eur J Hortic Sci. 2019;84(6):343–349.

[12] Tandonnet JP, Marguerit E, Cookson SJ, Ollat N. Genetic architecture of aerial and root traits in field-grown grafted grapevines is largely independent. Theor Appl Genet. 2018;131(4):903–915. 29305700 10.1007/s00122-017-3046-6

[13] Duchêne E, Dumas V, Jaegli N, Merdinoglu D. Deciphering the ability of different grapevine genotypes to accumulate sugar in berries. Aust J Grape Wine Res. 2012;18(3):319–328.

[14] Kliewer WM, Dokoozlian NK. Leaf area/crop weight ratios of grapevines: Influence on fruit composition and wine quality. Am J Enol Vitic. 2005;56(2):170–181.

[15] Parker AK. Reduced grapevine canopy size post-flowering via mechanical trimming alters ripening and yield of 'Pinot noir'. Vitis. 2016;55:1–9.

[16] Parker AK, Hofmann RW, van Leeuwen C, McLachlan ARG, Trought MCT. Leaf area to fruit mass ratio determines the time of veraison in sauvignon blanc and pinot noir grapevines. Aust J Grape Wine Res. 2014;20(3):422–431.

[17] Frioni T, Acimovic D, VanderWeide J, Tombesi S, Palliotti A, Gatti M, Poni S, Sabbatini P. Whole-canopy source-sink balance at bloom dictates fruit set in cv. Pinot noir subjected to early leaf removal. Am J Enol Vitic. 2019;70(4):411–419.

[18] Lopez-Urrea R, Montoro A, Manas F, Lopez-Fuster P, Fereres E. Evapotranspiration and crop coefficients from lysimeter measurements of mature 'Tempranillo' wine grapes. Agric Water Manag. 2012;112(3-4):13–20.

[19] Austin CN, Grove GG, Meyers JM, Wilcox WF. Powdery mildew severity as a function of canopy density: Associated impacts on sunlight penetration and spray coverage. Am J Enol Vitic. 2011;62(1):23–31.

[20] Roman C. Pesticide dose adjustment in fruit and grapevine orchards by DOSA3D: Fundamentals of the system and on-farm validation. Sci Total Environ. 2022;808:152158. 34871680 10.1016/j.scitotenv.2021.152158

[21] Vélez S, Poblete-Echeverría C, Rubio JA, Vacas R, Barajas E. Estimation of Leaf Area Index in vineyards by analysing projected shadows using UAV imagery. OENO One. 2021;55(4):159–180.

[22] Weiss M, Baret F. Using 3D point clouds derived from UAV RGB imagery to describe vineyard 3D macro-structure. Remote Sens. 2017;9(2):111.

[23] López-Lozano R. Tecnologías de información geográfica en la cartografía de parámetros biofísicos de parcelas de maíz y viña para agricultura de precisión [thesis]. Universidad de Zaragoza; 2008.

[24] Zarco-Tejada PJ et al. Assessing vineyard condition with hyperspectral indices: Leaf and canopy reflectance simulation in a row-structured discontinuous canopy. Remote Sens Environ. 2005;99(3):271–287.

[25] Diago M-P, Correa C, Millán B, Barreiro P, Valero C, Tardaguila J. Grapevine yield and leaf area estimation using supervised classification methodology on RGB images taken under field conditions. Sensors. 2012;12(12):16988–17006. 23235443 10.3390/s121216988 PMC3571822

[26] Diago MP, Tardaguila J, Barrio I, Fernández-Novales J. Combination of multispectral imagery, environmental data and thermography for on-the-go monitoring of the grapevine water status in commercial vineyards. Eur J Agron. 2022;140:126586.

[27] Tosin R. Estimation of grapevine predawn leaf water potential based on hyperspectral reflectance data in Douro wine region. Vitis. 2020;59:9–18.

[28] Wei HE, Grafton M, Bretherton M, Irwin M, Sandoval E. Evaluation of point hyperspectral reflectance and multivariate regression models for grapevine water status estimation. Remote Sens. 2021;13(16):3198.

[29] Oerke EC, Herzog K, Toepfer R. Hyperspectral phenotyping of the reaction of grapevine genotypes to Plasmopara viticola. J Exp Bot. 2016;67(18):5529–5543. 27567365 10.1093/jxb/erw318

[30] Gangl H, Tiefenbrunner M, Leitner G, Tiefenbrunner I, Tiefenbrunner W. The use of drones to detect and quantify grapevine diseases (bacterioses and viroses) in the vineyard. Mitteilungen Klosterneuburg. 2021;71:1–17.

[31] Nagi R, Tripathy SS. Severity estimation of grapevine diseases from leaf images using fuzzy inference system. Agric Res. 2021;11(12):112–122.

[32] Coupel-Ledru A, Lebon É, Christophe A, Doligez A, Cabrera-Bosquet L, Péchier P, Hamard P, This P, Simonneau T. Genetic variation in a grapevine progeny (*Vitis vinifera* L. cvs GrenachexSyrah) reveals inconsistencies between maintenance of daytime leaf water potential and response of transpiration rate under drought. J Exp Bot. 2014;65(21):6205–6218. 25381432 10.1093/jxb/eru228 PMC4223985

[33] Rist F, Schwander F, Richter R, Mack J, Schwandner A, Hausmann L, Steinhage V, Töpfer R, Herzog K. Relieving the phenotyping bottleneck for grape bunch architecture in grapevine breeding research: Implementation of a 3D-based phenotyping approach for quantitative trait locus mapping. Horticulturae. 2022;8(10):907.

[34] Jin S, Sun X, Wu F, Su Y, Li Y, Song S, Xu K, Ma Q, Baret F, Jiang D, et al. Lidar sheds new light on plant phenomics for plant breeding and management: Recent advances and future prospects. ISPRS J Photogramm Remote Sens. 2021;171:202–223.

[35] Lopez-Lozano R, Baret F. 2D approximation of realistic 3D vineyard row canopy representation for light interception (fIPAR) and light intensity distribution on leaves (LIDIL). Eur J Agron. 2011;35(3):171–183.

[36] Arnó J, Escolà A, Vallès JM, Llorens J, Sanz R, Masip J, Palacín J, Rosell-Polo JR. Leaf area index estimation in vineyards using a ground-based LiDAR scanner. Precis Agric. 2013;14(2):290–306.

[37] Bailey BN, Mahaffee WF. Rapid measurement of the three-dimensional distribution of leaf orientation and the leaf angle probability density function using terrestrial LiDAR scanning. Remote Sens Environ. 2017;194:63–76.

[38] del Campo Sanchez A, Moreno M, Ballesteros R, Hernandez-Lopez D. Geometric characterization of vines from 3D point clouds obtained with laser scanner systems. Remote Sens. 2019;11(20):2365.

[39] Kronenberg L, Yates S, Boer MP, Kirchgessner N, Walter A, Hund A. Temperature response of wheat affects final height and the timing of stem elongation under field conditions. J Exp Bot. 2021;72(2):700–717. 33057698 10.1093/jxb/eraa471 PMC7853599

[40] Heun JT, Attalah S, French AN, Lehner KR, McKay JK, Mullen JL, Ottman MJ, Andrade-Sanchez P. Deployment of Lidar from a ground platform: Customizing a low-cost, information-rich and user-friendly application for field Phenomics research. Sensors. 2019;19(24):5358. 31817334 10.3390/s19245358 PMC6960510

[41] Coupel-Ledru A, Pallas B, Delalande M, Segura V, Guitton B, Muranty H, Durel CE, Regnard JL, Costes E. Tree architecture, light interception and water-use related traits are controlled by different genomic regions in an apple tree core collection. New Phytol. 2022;234(1):209–226. 35023155 10.1111/nph.17960 PMC9305758

[42] Taskos DG, Koundouras S, Stamatiadis S, Zioziou E, Nikolaou N, Karakioulakis K, Theodorou N. Using active canopy sensors and chlorophyll meters to estimate grapevine nitrogen status and productivity. Precis Agric. 2015;16(1):77–98.

[43] Lorenz DH, Eichhorn KW, Bleiholder H, Klose R, Meier U, Weber E. Growth stages of the grapevine: Phenological growth stages of the grapevine (*Vitis vinifera* L. *ssp. vinifera*)—Codes and descriptions according to the extended BBCH scale. Aust J Grape Wine Res. 1995;1(2):100–103.

[44] Carbonneau A. L'exposition utile du feuillage: Définition du potentiel du système de conduite. In: *Systèmes de conduite de la vigne et mécanisation*. Paris: OIVV; 1989. p. 13–33.

[45] Moreno H, Valero C, Bengochea-Guevara JM, Ribeiro Á, Garrido-Izard M, Andújar D. On-ground vineyard reconstruction using a LiDAR-based automated system. Sensors. 2020;20(4):1102. 32085436 10.3390/s20041102 PMC7070798

[46] R Core Team. R Foundation for Statistical Computing, Vienna, Austria; 2021.

[47] Elshire RJ, Glaubitz JC, Sun Q, Poland JA, Kawamoto K, Buckler ES, Mitchell SE. A robust, simple genotyping-by-sequencing (GBS) approach for high diversity species. PLoS One. 2011;6(5):e19379. 21573248 10.1371/journal.pone.0019379 PMC3087801

[48] Velt A, Frommer B, Blanc S, Holtgräwe D, Duchêne É, Dumas V, Grimplet J, Hugueney P, Kim C, Lahaye M, et al. An improved reference of the grapevine genome reasserts the origin of the PN40024 highly-homozygous genotype. G3 (Bethesda). 2023;13(5):jkad067. 36966465 10.1093/g3journal/jkad067 PMC10151409

[49] Li H. Aligning sequence reads, clone sequences and assembly contigs with BWA-MEM. arXiv (2013).

[50] Catchen J, Hohenlohe PA, Bassham S, Amores A, Cresko WA. Stacks: An analysis tool set for population genomics. Mol Ecol. 2013;22(11):3124–3140. 23701397 10.1111/mec.12354 PMC3936987

[51] Rastas P. Lep-MAP3: Robust linkage mapping even for low-coverage whole genome sequencing data. Bioinformat. 2017;33(23):3726–3732. 10.1093/bioinformatics/btx494 29036272

[52] Broman KW, Wu H, Sen S, Churchill GA. R/QTL: QTL mapping in experimental crosses. Bioinformat. 2003;19(7):889–890. 10.1093/bioinformatics/btg112 12724300

[53] Velt A. An improved reference of the grapevine genome supports reasserting the origin of the PN40024 highly-homozygous genotype. bioRxiv. 2022;2022.2012.2021.521434. 10.1093/g3journal/jkad067 PMC10151409 36966465

[54] Ouellette LA, Reid RW, Blanchard SG, Brouwer CR. LinkageMapView—Rendering high-resolution linkage and QTL maps. Bioinformat. 2017;34(2):306–307. 10.1093/bioinformatics/btx576 PMC5860205 28968706

[55] Pimont F, Soma M, Dupuy J-L. Accounting for wood, foliage properties, and laser effective footprint in estimations of leaf area density from Multiview-LiDAR data. Remote Sens. 2019;11(13):1580.

[56] Rançon F, Keresztes B, Deshayes A, Tardif M, Abdelghafour F, Fontaine G, da Costa JP, Germain C. Designing a proximal sensing camera acquisition system for vineyard applications: Results and feedback on 8 years of experiments. Sensors. 2023;23(2):847. 36679645 10.3390/s23020847 PMC9865571

[57] Rose JC, Kicherer A, Wieland M, Klingbeil L, Töpfer R, Kuhlmann H. Towards automated large-scale 3D phenotyping of vineyards under field conditions. Sensors. 2016;16(12):2136. 27983669 10.3390/s16122136 PMC5191116

[58] Duchêne E, Monamy C, Langellier F, Jaegli N, Salber R, Meluc D, Panigai L. Incidence au vignoble des conditions de maturation Sur l'élaboration du rendement au cours de l'année suivante. J Int Sci Vigne Vin. 2003;37(2):103–116.

[59] Anastasiou E, Balafoutis A, Theocharis S, Theodorou N, Koundouras S, Fountas S. Assessment of laser scanner use under different settings in two differently managed vineyards for estimating pruning wood parameters. AgriEngineering. 2022;4(3):733–746.

[60] Kicherer A, Klodt M, Sharifzadeh S, Cremers D, Töpfer R, Herzog K. Automatic image-based determination of pruning mass as a determinant for yield potential in grapevine management and breeding. Aust J Grape Wine Res. 2017;23(1):120–124.

[61] Millan B, Diago MP, Aquino A, Palacios F, Tardaguila J. Vineyard pruning weight assessment by machine vision: Towards an on-the-go measurement system. OENO One. 2019;53(2):333–345.

[62] Coupel-Ledru A, Pallas B, Delalande M, Boudon F, Carrié E, Martinez S, Regnard JL, Costes E. Multi-scale high-throughput phenotyping of apple architectural and functional traits in orchard reveals genotypic variability under contrasted watering regimes. Hortic Res. 2019;6:52. 31044079 10.1038/s41438-019-0137-3 PMC6491481

[63] Keightley KE, Bawden GW. 3D volumetric modeling of grapevine biomass using tripod LiDAR. Comput Electron Agric. 2010;74(2):305–312.

[64] Arnó J, Escolà A, Vallès JM, Llorens J, Sanz R, Masip J, Palacín J, Rosell-Polo JR. 2012. Leaf area index estimation in vineyards using a ground-based LiDAR scanner. Precis Agric. 2012;14(3):290-306.

[65] Alahakoon D, Fennell A, Helget Z, Bates T, Karn A, Manns D, Mansfield AK, Reisch BI, Sacks G, Sun Q, et al. Berry anthocyanin, acid, and volatile trait analyses in a grapevine-interspecific F2 population using an integrated GBS and rhAmpSeq genetic map. Plants (Basel). 2022;11(5):696. 35270166 10.3390/plants11050696 PMC8912348

[66] Possamai T, Wiedemann-Merdinoglu S, Merdinoglu D, Migliaro D, de Mori G, Cipriani G, Velasco R, Testolin R. Construction of a high-density genetic map and detection of a major QTL of resistance to powdery mildew (*Erysiphe necator* Sch.) in Caucasian grapes (*Vitis vinifera *L.). BMC Plant Biol. 2021;21(1):528. 34763660 10.1186/s12870-021-03174-4 PMC8582213

[67] Rubio B, Lalanne-Tisné G, Voisin R, Tandonnet JP, Portier U, van Ghelder C, Lafargue M, Petit JP, Donnart M, Joubard B, et al. Characterization of genetic determinants of the resistance to phylloxera, *Daktulosphaira vitifoliae*, and the dagger nematode *Xiphinema index* from muscadine background. BMC Plant Biol. 2020;20(1):213. 32398088 10.1186/s12870-020-2310-0 PMC7218577

[68] Yin L, Karn A, Cadle-Davidson L, Zou C, Underhill A, Atkins P, Treiber E, Voytas D, Clark M. Fine mapping of leaf Trichome density revealed a 747-kb region on chromosome 1 in cold-hardy hybrid wine grape populations. Front Plant Sci. 2021;12: Article 587640. 33746993 10.3389/fpls.2021.587640 PMC7965957

[69] Bert PF, Bordenave L, Donnart M, Hévin C, Ollat N, Decroocq S. Mapping genetic loci for tolerance to lime-induced iron deficiency chlorosis in grapevine rootstocks (*Vitis sp*.). Theor Appl Genet. 2013;126(2):451–473. 23139142 10.1007/s00122-012-1993-5

[70] Guillaumie S, Decroocq S, Ollat N, Delrot S, Gomès E, Cookson SJ. Dissecting the control of shoot development in grapevine: Genetics and genomics identify potential regulators. BMC Plant Biol. 2020;20(1):43. 31996141 10.1186/s12870-020-2258-0 PMC6988314

[71] de Badts X, Dumas V, Jaegli N, Ley L, Merdinoglu D, Duchêne E. Integrating spatial variations in the vineyard to enhance quantitative trait locus (QTL) detection. Acta Hortic. 2019;1248(215-220):10.

