# Supplementary material for: LiDAR Is Effective in Characterizing Vine Growth and Detecting Associated Genetic Loci
Source: Plant Phenomics. 2023 Nov 17;5:0116. doi: 10.34133/plantphenomics.0116 (PMC10655830; doi:10.34133/plantphenomics.0116)
Supplement: Supplementary 1 — Fig. S1 Tables S1 to S5 [file plantphenomics.0116.f1.docx]

**Supplementary data**

Supplementary Table S1: Analysis of variance the relationship between apparent canopy volumes estimated with LiDAR sensors and exposed leaf areas for the two vintages. The model tested with the R procedure glm was "ELA ~ LiDARsummer * Year"

| Coefficients | Estimate | Std. Error | t value | Pr(>\|t\|) |
| --- | --- | --- | --- | --- |
| Intercept | 19.95 | 2.260e+00 | 8.828 | **< 2e-16** |
| LiDARsummer | 1.316e-04 | 6.135e-06 | 21.448 | **< 2e-16** |
| Year2021 | 4.97 | 3.203e+00 | 1.552 | 0.121643 |
| LiDARsummer:Year2021 | 3.426e-05 | 9.596e-06 | 3.570 | **0.000407** |

Supplementary Table S2: Analysis of variance the relationship between apparent wood volumes estimated with LiDAR sensors and pruning weights for the two vintages. The model tested with the R procedure glm was "PW ~ LiDAR_WoodApparentVolume * Year"

| Coefficients | Estimate | Std. Error | t value | Pr(>\|t\|) |
| --- | --- | --- | --- | --- |
| Intercept | 0.002305 | 0.047605 | 0.048 | 0.961 |
| LiDAR_WoodApparentVolume | 0.194185 | 0.012808 | 15.161 | **< 2e-16** |
| Year2021 | 0.044076 | 0.066330 | 0.664 | 0.507 |
| LiDAR_WoodApparentVolume:Year2021 | -0.068427 | 0.015704 | -4.357 | **1.69e-05** |

Supplementary Table S3: coefficients of determination R^2^ between variables. The relationships were positive and all statistically significant at p<10^-9^, unless specified.

|  | Exposed leaf area | | Apparent canopy volume (LiDAR) | | Pruning Weight | | Apparent wood volume (LiDAR) | | Chlorophyll content  2020 |
| --- | --- | --- | --- | --- | --- | --- | --- | --- | --- |
|  | 2020 | 2021 | 2020 | 2021 | 2020 | 2021 | 2020 | 2021 |  |
| Exposed leaf area 2021 | 0.421 |  |  |  |  |  |  |  |  |
| Apparent canopy volume (LiDAR) summer 2020 | 0.790 | 0.428 |  |  |  |  |  |  |  |
| Apparent canopy volume (LiDAR) summer 2021 | 0.424 | 0.689 | 0.523 |  |  |  |  |  |  |
| Pruning Weight 2020 | 0.269 | 0.187 | 0.316 | 0.190 |  |  |  |  |  |
| Pruning Weight 2021 | 0.183 | 0.238 | 0.275 | 0.274 | 0.559 |  |  |  |  |
| Apparent wood volume (LiDAR) 2020 | 0.357 | 0.286 | 0.432 | 0.303 | 0.590 | 0.415 |  |  |  |
| Apparent wood volume (LiDAR) 2021 | 0.352 | 0.531 | 0.460 | 0.590 | 0.284 | 0.456 | 0.484 |  |  |
| Chlorophyll content 2020 | 0.02*  (R = -0.142) | 0.01^ns^ | 0.000^ns^ | 0.004^ns^ | 0.012^ns^ | 0.025* | 0.005^ns^ | 0.002^ns^ |  |
| Chlorophyll content 2021 | 0.009^ns^ | 0.007^ns^ | 0.022* | 0.007^ns^ | 0.017^ns^ | 0.032* | 0.006^ns^ | 0.02* | 0.201 |

*= 0.01<p<0.05, ^ns^ : not significant

Supplementary Table S4: Shapiro-Wilk test for normality

| Trait | Vintage | W | P-value |
| --- | --- | --- | --- |
| Exposed leaf area | 2019 | 0.97007 | 0.0007384*** |
|  | 2020 | 0.98811 | 0.08709 |
|  | 2021 | 0.98044 | 0.005931** |
| Chlorophyll content | 2020 | 0.98863 | 0.1046 |
|  | 2021 | 0.98814 | 0.08156 |
| Apparent canopy volume at véraison | 2020 | 0.99469 | 0.764 |
|  | 2021 | 0.99303 | 0.5831 |
| Voxel number before pruning (February) | 2020 | 0.9914 | 0.294 |
|  | 2021 | 0.99465 | 0.6812 |
| Voxel number after pruning (March) | 2020 | 0.99022 | 0.2012 |
|  | 2021 | 0.96814 | 0.0001359*** |
| Apparent pruning wood volume | 2020 | 0.99085 | 0.2465 |
|  | 2021 | 0.99545 | 0.8008 |
| Pruning fresh weight | 2020 | 0.9907 | 0.2347 |
|  | 2021 | 0.98707 | 0.06406 |

Supplementary Table S5: Main features of the genetic maps

|  | Divona | IJ119 | Consensus |
| --- | --- | --- | --- |
| Nb of genotypes | 249 | 249 | 249 |
| Nb of linkage groups | 19 | 19 | 19 |
| Number of markers | 20134 | 19935 | 28274 |
| Number of non-redundant markers | 1803 | 1582 | 4773 |
| Length (cM) | 1177 | 1110.1 | 1017.3 |
| Mean distance (cM) | 0.1 | 0.1 | 0.1 |
| Max. distance (cM) | 31.8 | 30.6 | 17.8 |

Supplementary Figure S1: Comparison between genetic and physical marker order in the linkage groups of three constructed maps. The x-axis indicates the position of the markers in the reference genome. Each dot indicates a marker, and its color indicates the map: red for the IJ119 map, blue for the Divona map, and green for the consensus map
